# Alignment-Free Population Genomics: An Efficient Estimator of Sequence Diversity

**DOI:** 10.1534/g3.112.002527

**Published:** 2012-08-01

**Authors:** Bernhard Haubold, Peter Pfaffelhuber

**Affiliations:** * Department of Evolutionary Genetics, Max Planck Institute for Evolutionary Biology, 24306 Plön, Germany; † Mathematical Stochastics, Mathematical Institute, Albert-Ludwigs University, 79085 Freiburg, Germany

**Keywords:** genetic diversity, alignment-free, maximum-likelihood, *Drosophila*, match length distribution

## Abstract

Comparative sequencing contributes critically to the functional annotation of genomes. One prerequisite for successful analysis of the increasingly abundant comparative sequencing data is the availability of efficient computational tools. We present here a strategy for comparing unaligned genomes based on a coalescent approach combined with advanced algorithms for indexing sequences. These algorithms are particularly efficient when analyzing large genomes, as their run time ideally grows only linearly with sequence length. Using this approach, we have derived and implemented a maximum-likelihood estimator of the average number of mismatches per site between two closely related sequences, *π*. By allowing for fluctuating coalescent times, we are able to improve a previously published alignment-free estimator of *π*. We show through simulation that our new estimator is fast and accurate even with moderate recombination (*ρ* ≤ *π*). To demonstrate its applicability to real data, we compare the unaligned genomes of *Drosophila persimilis* and *D. pseudoobscura*. In agreement with previous studies, our sliding window analysis locates the global divergence minimum between these two genomes to the pericentromeric region of chromosome 3.

A central goal of modern biology is to explain in molecular detail the relationship between genotypes and phenotypes. The success of this research agenda depends on intimate knowledge of phenotypic and genotypic diversity collected from a wide variety of organisms. Historically, knowledge about genotypic diversity has been much scarcer than about phenotypic variation. This is now changing rapidly with several projects under way to sequence the complete genomes of 1000 individuals belonging to the same species.

Quantifying the genetic diversity from such sequence data is conceptually simple: after assembly, align the sequences and calculate one or more of several well-known estimators of genetic diversity (Wakeley 2009, ch. 4). However, calculating alignments between genomes can be cumbersome for two reasons. First, the sequencing phase of genome projects typically results in hundreds to thousands of contigs rather than chromosome-length assemblies. Second, genome rearrangements disrupt the synteny implicit in many alignment procedures.

One way to avoid assembling and aligning sets of long sequences is to restrict the analysis to mapping the sequencing reads onto an existing reference genome. Still, the sheer superabundance of sequencing data has motivated the development of new computational approaches even for dealing with the comparatively simple task of mapping short reads. Some of the most efficient solutions to the mapping problem currently available are implemented in programs like bwa (Li and Durbin 2009), bowtie (Langmead *et al.* 2009), and soap (Li *et al.* 2009), which are based on recent advances in algorithms for indexing long sequences (Puglisi *et al.* 2007). Such algorithms are optimal in the strong sense that computation of the underlying indexes can be achieved in time that grows only linearly with the size of the input data.

To take advantage of these new algorithms in population genetics, we have been working on methods for quantifying genetic diversity based on string indexing. The central idea here is that of a shortest unique substring or *shustring* (Haubold *et al.* 2005). When considering a query sequence, *Q*, and a subject sequence, *S*, a shustring starting at position *i* in *Q* is the shortest substring *Q*[*i*..*i* + *x* – 1] that does not appear in *S*, and we say that such a shustring has length *x*. The average length of shustrings decreases with diversity, *i.e.*, if the shustrings are long, *S* and *Q* are closely related, and if the shustrings are short, *S* and *Q* are more diverged.

This notion can be made precise by deriving an estimator of the substitution rate based on the distribution of shustring lengths (Haubold *et al.* 2009). Domazet-Lošo and Haubold (2009) implemented this estimator in a program for quickly clustering genomes from organisms as diverse as HIV with 9 kb genomes and *Drosophila* with 170 Mb genomes. They also generalized the computation of global evolutionary distances to the detection of local homology between a query and a set of subject sequences (Domazet-Lošo and Haubold 2011b). This is particularly useful for the detection of horizontal gene transfer among bacteria (Domazet-Lošo and Haubold 2011a).

Recently Haubold *et al.* (2011) have begun to develop shustring-based estimators for population genetics. A first simple, but powerful, result of this work is that the expected number of pairwise mismatches, *π*, is approximately equal to the inverse of the average shustring length. Haubold *et al.* (2011) designated this estimator ${\overset{⁁}{\pi }}_{\text{m}}$and used it to locate the divergence minimum between *Drosophila simulans* and *D. sechellia* to a region centered on the gene pickpocket (*ppk*). This gene may be involved in the characteristic preference of *D. sechellia* larvae for the fruit of *Morinda citrifolia*, which is toxic to other *Drosophila* (Dworkin and Jones 2009). It took less than 23 min on a single AMD Opteron 2.3 GHz processor to calculate the local divergence along the complete genomes of *D. simulans* and *D. sechellia* (Haubold *et al.* 2011). Moreover, the genome of *D. sechellia* consisted of 14,730 contigs, which would normally complicate sequence analysis. However, the computation of shustring lengths does not require synteny and can therefore be applied to unordered contigs.

${\overset{⁁}{\pi }}_{\text{m}}$ is easy to compute and is accurate in the absence of recombination. However, Haubold *et al.* (2011) already pointed out that ${\overset{⁁}{\pi }}_{\text{m}}$ is downward biased if *Q* and *S* have undergone recombination. Intuitively, this observation can be understood from the well-known fact that SNPs tend to cluster along the genome in the presence of recombination. We therefore report here the replacement of ${\overset{⁁}{\pi }}_{\text{m}}$ by a maximum-likelihood estimator, ${\overset{⁁}{\pi }}_{\text{d}}$, based on the full distribution of shustring lengths (subscript d for distribution). In contrast to ${\overset{⁁}{\pi }}_{\text{m}}$ (subscript m for mean), which rests on the assumption of a constant coalescence time across the two sequences compared, ${\overset{⁁}{\pi }}_{\text{d}}$ allows local fluctuations in coalescence times. This makes ${\overset{⁁}{\pi }}_{\text{d}}$ much more robust against recombination than ${\overset{⁁}{\pi }}_{\text{m}}$ but still simple enough to allow efficient repeated computation in sliding window analyses.

In the following sections, we derive ${\overset{⁁}{\pi }}_{\text{d}}$ and test our implementation of it, pid, through simulation. We then apply pid to two pairs of complete *Drosophila* genomes. The first is an aligned pair taken from the Drosophila Population Genomics Project to allow comparison between *π* and ${\overset{⁁}{\pi }}_{\text{d}}$. The second pair consists of the unaligned genomes of the closely related species *D. pseudoobscura* and *D. persimilis*, in which *D. persimilis* consists of 12,838 contigs. We focus our analysis on regions of low divergence. These are singled out in many studies as candidate regions affected by important evolutionary events, including introgression and selective sweeps.

## Approach and Data

### Shortest unique substrings

Let *Q* and *S* be two DNA sequences called *query* and *subject* of lengths 2ℓ*_Q_* and 2ℓ*_S_*, respectively. In our analysis, we use both the forward and reverse strands; hence, the factors 2 in the lengths of the sequences. A shustring of sequence *Q* starting at position 1 ≤ *i* ≤ 2ℓ*_Q_* is the shortest substring that differs from substrings starting at any position 1 ≤ *i*′ ≤ 2ℓ*_s_* in *S*. We denote the lengths of shustrings starting at positions *i*, *i*′ in sequences *Q*, *S* by ${\tilde{X}}_{i,i^{\prime }}$. Put more formally, ${\tilde{X}}_{i,i^{\prime }}=x$ if positions *i*, …, *i* + *x* – 2 in *Q* and *i*′, …, *i*′ + *x* – 2 in *S* are identical but the nucleotide *i* + *x* – 1 in *Q* differs from nucleotide *i*′ + *x* – 1 in *S*. Then, the shustring starting at position *i* in *Q* is given by ${\tilde{X}}_{i}^{*}:=\max_{i^{\prime }}{\tilde{X}}_{i,i^{\prime }}.$

This definition is only useful if *i* is not too close to 2ℓ*_S_* or *i*′ is not too close to 2ℓ*_S_* because the shustring starting at *i* can at most be of length 2ℓ*_S_* – *i*. Therefore, we use $X_{i,i^{\prime }}=\min\left({\tilde{X}}_{i,i^{\prime }},\left(2\ell_{S}-i\right),\left(2\ell_{Q}-i^{\prime }\right)\right)$ and

$$X_{i}^{*}:=\underset{i^{\prime }}{\max}X_{i,i^{\prime }}.$$

Our approach presented below works as long as we can neglect edge effects, *i.e.*, ${\tilde{X}}_{i}^{*}=X_{i}^{*}$ for most *i*. In practice, we simplify the analysis by concatenating all query contigs into one sequence and all subject sequences into another sequence. This means that shustrings can span contig borders but are cut off beyond a border after a—usually short—run of random matches. A large number of contigs will therefore lead to an excess of short shustrings and a corresponding overestimation of *π*.

### Determining the shustring length distribution from data

**Figure 1:**
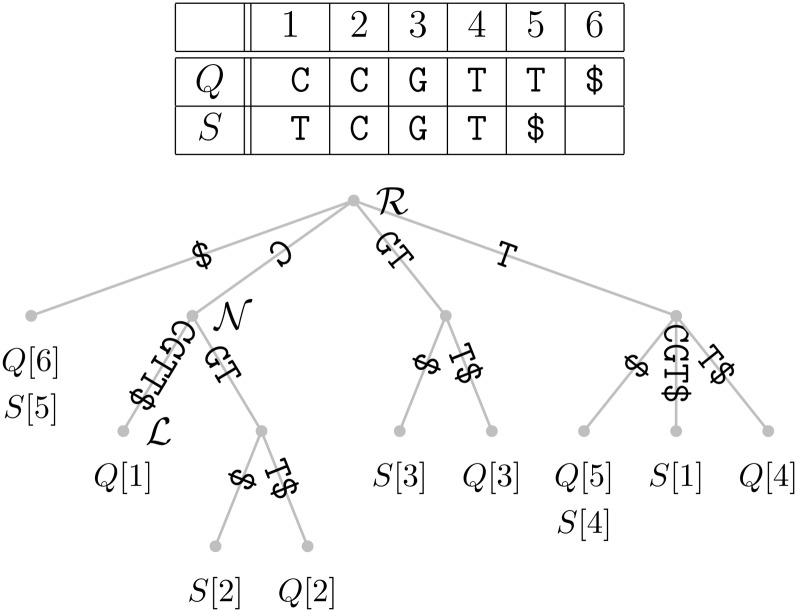
Two sequences, *Q* and *S* (top), and the corresponding suffix tree (bottom). ℛ, the root; 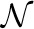, an internal node; ℒ, a leaf.

The input for the computation of shustring lengths consists of two sequences, one query, and one subject. These have been obtained by concatenating a potentially large number of contigs. For the purposes of this exposition, let the query *Q* = CCGTT and the subject *S* = TCGT. A *suffix* is a string that starts anywhere in *Q* or *S* and ends at the end. For example, TT is a suffix of *Q*. The first step in our analysis is to index all suffixes contained in *Q* and *S*. The resulting data structure is called a *suffix tree* and is shown in Figure 1 (Gusfield 1997). The defining feature of this tree is that the concatenated labels on the path from the root, ℛ, to a leaf labeled *Q*[*i*] spell out the suffix starting at position *i* in *Q*. For example, the path label of leaf ℒ is CCGTT$, which is the suffix starting at *Q*[1]. Notice the character $ that terminates *S* and *Q*. This ensures that no suffix of *Q* can simultaneously be a prefix of a different suffix of *Q*, a technicality that guarantees that every suffix is represented by a leaf in the tree. To look up the shustring starting at *Q*[1], we walk from ℒ toward ℛ until we find a node that contains a subject leaf in the subtree rooted on it. In our example, we find this node, 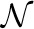, in one step. The path label of 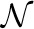, C, becomes the desired shustring when we extend it by one nucleotide to obtain CC. Our approach is centered on the distribution of the lengths of such shustrings starting at every position in *Q*. These can be looked up in a single traversal of the relevant suffix tree.

The speed of our method relies on the fact that suffix trees can be constructed in time that is linear in the number of nucleotides analyzed (Gusfield 1997). In practice, an explicit suffix tree uses too much memory for genomics applications. Instead, an abstraction of a suffix tree based on a suffix array is commonly used. This is an alphabetically ordered list of all suffices in a sequence. By traversing this simple data structure, the corresponding suffix tree can also be traversed (Abouelhoda *et al.* 2002).

### Derivation of ${\overset{⁁}{\pi }}_{\text{d}}$

We wish to know the distribution of shustring lengths as a function of the average number of differences per site. For the derivation of the shustring length distribution, we use the well-kown fact from coalescent theory that the time to the most recent common ancestor of two lineages is approximately exponential with expectation *N_e_*, where *N_e_* is the effective (haploid) population size (Hudson 1990).

First, we compute the distribution of *X_i_*_,_*_i_*, where we assume that position *i* in *Q* and *i* in *S* are homologous. The fact that our data are unaligned does not invalidate this assumption for the purpose of deriving ${\overset{⁁}{\pi }}_{\text{d}}$. We further assume that no recombination event falls between *i* and *i* + *X_i_*_,_*_i_* until *Q* and *S* coalesce in this genomic region. This is equivalent to assuming that mutation is more frequent than recombination. Moreover, we take *π*/*N_e_* as a proxy for the mutation probability per generation per site. Then we obtain for an exponentially distributed random variable *T* with expectation *N_e_*

(1) $$\mathbb{P}\left\{X_{i,i}\;>\;x\right\}=E\left[\mathbb{P}\left\{X_{i,i}\;>\;x|T\right\}\right]=E\left[e^{-\pi Tx/N_{e}}\right]=\frac{1}{1+\pi x}.$$

Second, we compute the distribution of *X_i_*_,_*_i_*_′_ for *i* ≠ *i*′. We assume that the two subsequences starting at *i* in *Q* and at *i*′ in *S* are random words with *GC*-content 2*p* and *AT*-content 1 – 2*p*. We know from equation 1 in Haubold *et al.* (2005) and equation 4 in Haubold *et al.* (2009) that

(2) $$\mathbb{P}\left\{\max_{i\neq i^{\prime }}X_{i,i^{\prime }}\leq x\right\}=\sum_{k=0}^{x}2^{x}\left(\begin{array}{c} x\\ k \end{array}\right)p^{k}{\left(\frac{1}{2}-p\right)}^{x-k}{\left(1-p^{k}{\left(\frac{1}{2}-p\right)}^{x-k}\right)}^{2\ell_{S}}=:w_{p,\ell_{S}}\left(x\right),$$

which for equiprobable nucleotides $\left(p=\frac{1}{4}\right)$ simplifies to

(3) $$\mathbb{P}\left\{\underset{i\neq i^{\prime }}{\max}X_{i,i^{\prime }}\;\leq \;x\right\}={\left(1-4^{-x}\right)}^{2\ell_{S}}.$$

In this case, the distribution of max*_i_*_≠_*_i_*_′_*X_i_*_,_*_i_*_′_ is concentrated around *x* ∼ log _4_(2ℓ*_S_*). By combining Equations 1 and 2, we obtain

(4) $$\mathbb{P}\left\{X_{i}^{*}\leq x\right\}=w_{p,\ell_{S}}\left(x\right)\frac{\pi x}{1+\pi x},$$

and

(5) $$p_{\pi }\left(x\right):=\mathbb{P}\left\{X_{i}^{*}=x\right\}=w_{p,\ell_{S}}\left(x\right)\frac{\pi x}{1+\pi x}-w_{p,\ell_{S}}\left(x-1\right)\frac{\pi \left(x-1\right)}{1+\pi \left(x-1\right)}.$$

As explained in the previous section, we can observe

f(1),f(2),…f(ξ),

where *f*(*x*) is the absolute number of shustrings of length *x* for a pair of sequences and *ξ* is the length of the longest shustring. Now we assume that *f*(*x*) is the realization of a Poisson-distributed random variable with parameter $2p_{\pi }\left(x\right)\ell_{Q}^{*}$ and that *f*(1), *f*(2), … are independent. We can then readily compute the log-likelihood

$$\begin{array}{c} \log L\left(\pi |f\left(1\right),f\left(2\right),\ldots ,f\left(\xi \right)\right)=\sum_{x=1}^{\xi }-2p_{\pi }\left(x\right)\ell_{Q}+f\left(x\right)\log\left(2p_{\pi }\left(x\right)\ell_{Q}\right)-\log\left(f\left(x\right)!\right)\\ =\sum_{x=1}^{\xi }f\left(x\right)\log p_{\pi }\left(x\right)+C \end{array}$$

for some *C*, which does not depend on *π*. Hence, the maximum-likelihood estimator for *π*, ${\overset{⁁}{\pi }}_{\text{d}}$, is given by maximizing

(6) $$\sum_{x=1}^{\xi }f\left(x\right)\log p_{\pi }\left(x\right).$$

One often needs to compute ${\overset{⁁}{\pi }}_{\text{d}}$ repeatedly during a sliding window analysis, where an interval *Q*[*i*..*j*] is fixed with, say, *j* – *i* = 10^5^, *i.e.*, extends over 100 kb. Then, using *Q*[*i*, …, *j*] as the query and *S* as the subject, the above computations work as well, as we can still assume that every position in *Q*[*i*, …, *j*] has a homolog in *S*. Here, we observe *f*(1), *f*(2), … for the specific window *Q*[*i*, …, *j*], and maximize the likelihood as given in Equation 6.

A problem inherent in our method is that query windows without a full homolog in the subject sequence contain an excess of short random shustrings and are hence assigned too large a value of ${\overset{⁁}{\pi }}_{d}$. We mitigated this problem by applying the criterion that if in a window of length *l*_w_ the number of shustring peaks is greater than *l*_w_ × max(*π*), the window is deemed “missing data.” A shustring peak occurs at position *i* if the shustring length at position *i* – 1 is less than or equal to the shustring length at position *i*. This means that the shustring tracked at position *i* – 1 refers to a different SNP from the shustring tracked at position *i*. Using the simulations shown in Figure 4 to guide us, we set max(*π*) = 0.06, as the algorithm worked for *π* = 0.04 but not any more for *π* = 0.08.

### Implementation

We have implemented the calculation of ${\overset{⁁}{\pi }}_{\text{d}}$ in the program pid. The underlying suffix array computation is based on a software library by Manzini and Ferragina (2002). pid is available under the GNU General Public License from http://guanine.evolbio.mpg.de/pid/

This website hosts the C sources of the program and detailed user documentation.

### Data

The genome sequences of *D. melanogaster* strains RAL-365_1 and RAL-391_2 were downloaded from the Drosophila Population Genomics Project website (www.dpgp.org). For the alignment-free analysis, padding Ns were removed.

Whole-genome sequences of 21 *Drosophila* species were downloaded from the following three websites:

1. The genomes of *D. grimshawi*, *D. mojavensis*, *D. virilis*, *D. willistoni*, *D. persimilis*, *D. pseudoobscura*, *D. erecta*, *D. yakuba*, *D. melanogaster*, *D. sechellia*, *D. simulans*, and *D. ananassae* from the website of the 12 Drosophila species genome sequencing project (Drosophila 12 Genomes Consortium 2007) (rana.lbl.gov/drosophila/caf1/all_caf1.tar.gz).
2. The genomes of *D. bipectinata*, *D. kikkawai*, *D. elegans*, *D. ficusphila*, *D. rhopaloa*, *D. biarmipes*, and *D. takahashii*, which were sequenced at the Baylor College of Medicine (http://www.hgsc.bcm.tmc.edu/collaborations/insects/dros_modencode/GAsm/).
3. The genome of *D. santomea* sequenced by the Andolfatto lab (http://genomics.princeton.edu/AndolfattoLab/Dsantomea_genome.html).

Again, Ns were removed from the sequences of up to 23,004 contigs.

### Simulations

Pairs of query/subject sequences were generated using our program generateQuerySbjct, which is also available from the pid home page. generateQuerySbjct calls the coalescent simulation program ms (Hudson 2002) or macs (Chen *et al.* 2009), and converts the output using our program ms2dna (available from http://guanine.evolbio.mpg.de/bioBox).

### Phylogeny reconstruction

We applied the program kr (Domazet-Lošo and Haubold 2009) to estimate all pairwise distances between the 21 complete *Drosophila* genomes currently available. The resulting distance matrix was clustered using the neighbor-joining algorithm as implemented in neighbor, which is part of PHYLIP (Felsenstein 2005). The tree was midpoint-rooted using retree and drawn with drawgram, both also part of PHYLIP.

To compare this tree with the corresponding alignment-based phylogeny, we followed a study of *Drosophila* evolution centered on the *Amyrel* gene (Da Lage *et al.* 2007): we aligned the *Amyrel* sequences from each organism and computed the neighbor-joining tree using clustalw (Larkin *et al.* 2007). Rooting and drawing the tree was done as just described for the alignment-free cluster diagram.

## Results

**Figure 2:**
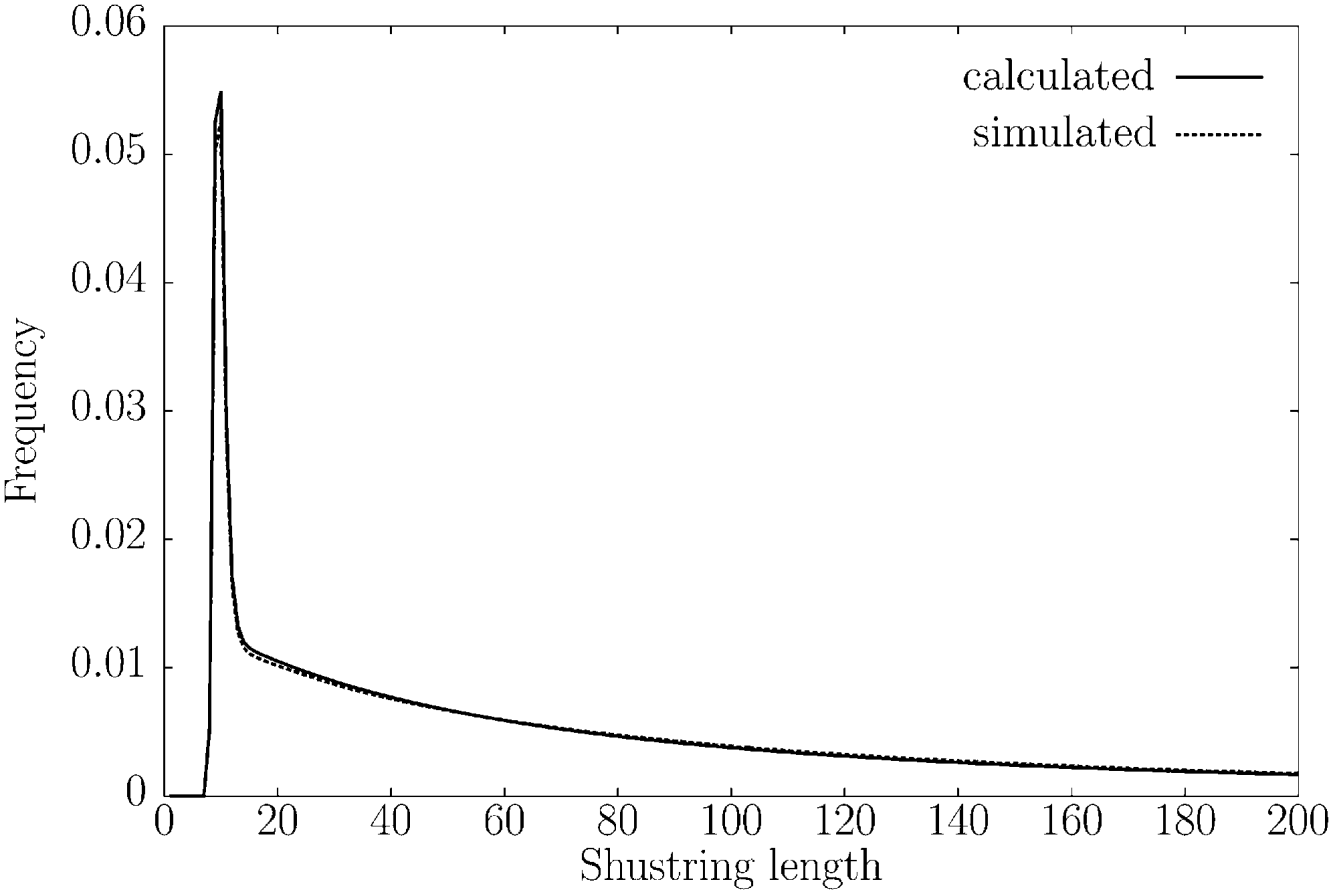
Comparison between the theoretical distribution of shustring lengths and the simulated distribution. *ρ* = 0.01, *π* = 0.01, and sequences were 100 kb long; simulations were averaged over 1000 iterations.

We started our investigation of the properties of ${\overset{⁁}{\pi }}_{\text{d}}$ by comparing the theoretical distribution of shustring lengths with that obtained through simulation. For the simulation, we generated 1000 pairs of DNA sequences of length 100 kb conditioned on *π* = 0.01 mismatches per position and a rate of recombination of *ρ* = 0.01. From this, we averaged the distribution of the shustring lengths. Figure 2 shows that this simulated distribution is closely approximated by the theoretical distribution. Notice also that the distribution of shustring lengths is strongly heavy tailed in the formal sense that *E*[X_i_^*^ – *x*|X_i_^*^ > *x*] = ∞; in particular, the shustring length distribution has no finite expectation.

**Figure 3:**
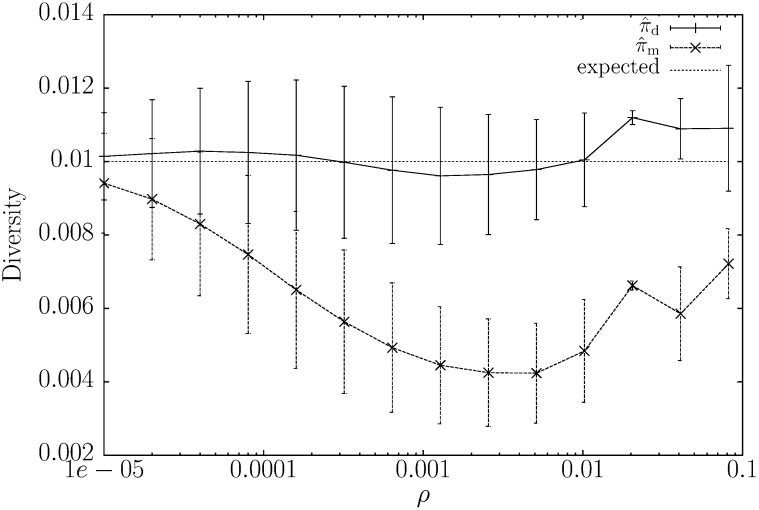
Comparison between our previous alignment-free estimator of genetic diversity, ${\overset{⁁}{\pi }}_{\text{m}}$, and our new estimator, ${\overset{⁁}{\pi }}_{\text{d}}$, as a function of *ρ*. Pairs of 100 kb sequences were simulated with *π* = 0.01 and data points are mean ± SD determined from 10,000 iterations.

To determine whether the theoretical shustring length distribution could be used to estimate *π*, we again simulated pairs of 100 kb DNA sequences at values of *ρ* ranging from 0 to 0.082, while keeping *π* = 0.01 constant. As Haubold *et al.* (2011) had reported before, the previous estimator, ${\overset{⁁}{\pi }}_{\text{m}}$, worked well for *ρ* = 0, but was strongly downward biased for *ρ* > 0 (Figure 3). In contrast, our new estimator, ${\overset{⁁}{\pi }}_{\text{d}}$, gives good results for *ρ* ≤ *π*. For larger values of *ρ*, it is biased upward (Figure 3).

**Figure 4:**
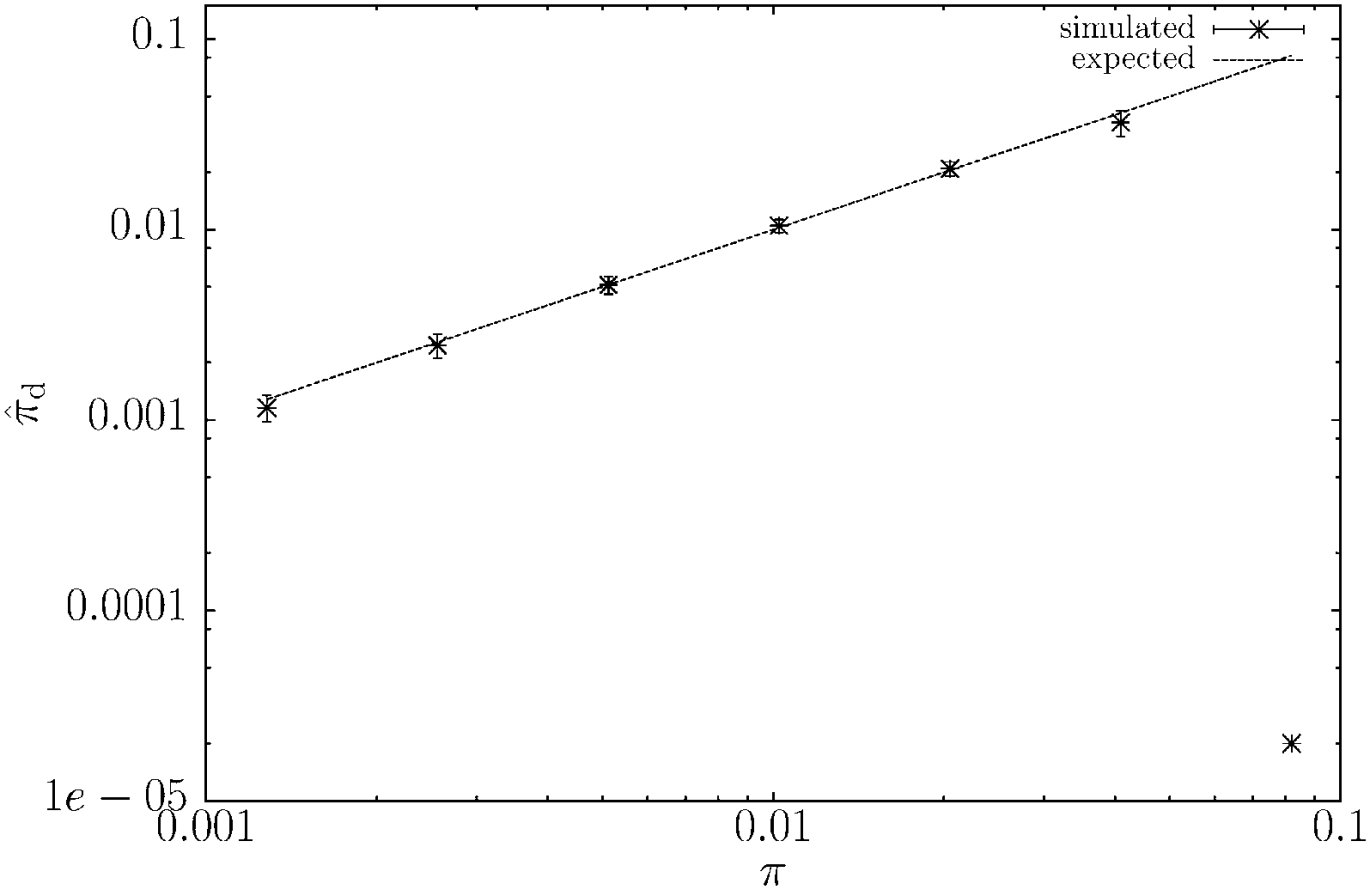
The new estimator of the number of pairwise mismatches, ${\overset{⁁}{\pi }}_{\text{d}}$, as a function of the number of pairwise mismatches, *π*. 10^4^ pairs of 100 kb sequences were simulated to compute mean ± SD. Here, we set *ρ* = 0.01 for all values of *π*.

Instead of varying *ρ* and keeping *π* constant, we also varied *π* while keeping *ρ* constant at 0.01. Figure 4 shows that for *π* ≤ 0.02 the estimates are very close to the true values. For more divergent sequences, ${\overset{⁁}{\pi }}_{\text{d}}$ becomes downward biased and then breaks down, as shown for *π* = 0.08.

**Figure 5:**
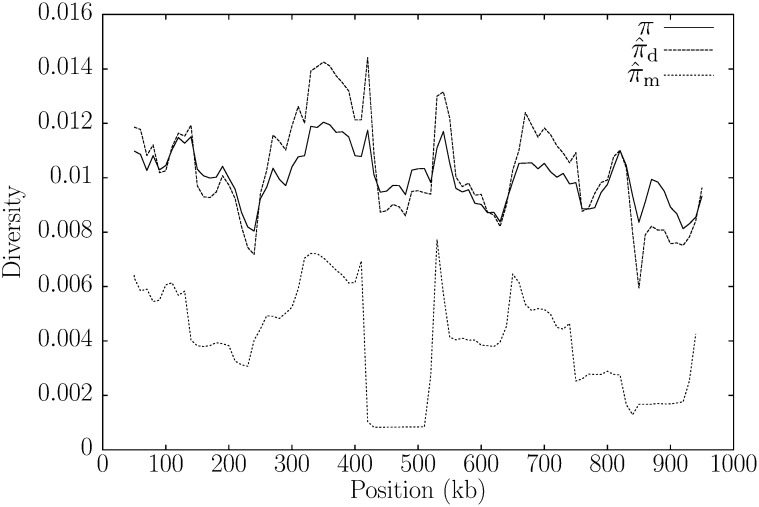
The values of the diversity measures *π*, ${\overset{⁁}{\pi }}_{\text{d}}$, and ${\overset{⁁}{\pi }}_{\text{m}}$ along a pair of simulated sequences 1 Mb long.

Up to now, we have estimated global values of *π*. However, it is often more interesting to study the local variation in *π* through a sliding window analysis. To investigate the suitability of ${\overset{⁁}{\pi }}_{\text{d}}$ for this, we simulated a 1 Mb sequence pair with *π* = *ρ* = 0.01. In Figure 5, 100 kb sliding windows of *π* are compared with ${\overset{⁁}{\pi }}_{\text{d}}$. Although ${\overset{⁁}{\pi }}_{\text{d}}$ tends to exaggerate the fluctuations of the *π* curve, it tracks it much more faithfully than ${\overset{⁁}{\pi }}_{\text{m}}$ and appears to be unbiased. This visual impression is corroborated by the averages of *π* and ${\overset{⁁}{\pi }}_{\text{d}}$, which are very similar (avg(*π*) = 1.003 × 10^−2^; $\text{avg}\left({\overset{⁁}{\pi }}_{\text{d}}\right)=1.032\times 10^{-2}$).

Sliding window analyses are only feasible if the statistic of interest can be computed efficiently. Our program pid took 9.1 sec on a single Intel Xeon 3 GHz CPU to analyze the 90 windows of 100 kb summarized in Figure 5. This is, of course, much slower than the computation of *π* given an alignment. But without an alignment, it is quick enough to analyze realistic data sets.

**Figure 6:**
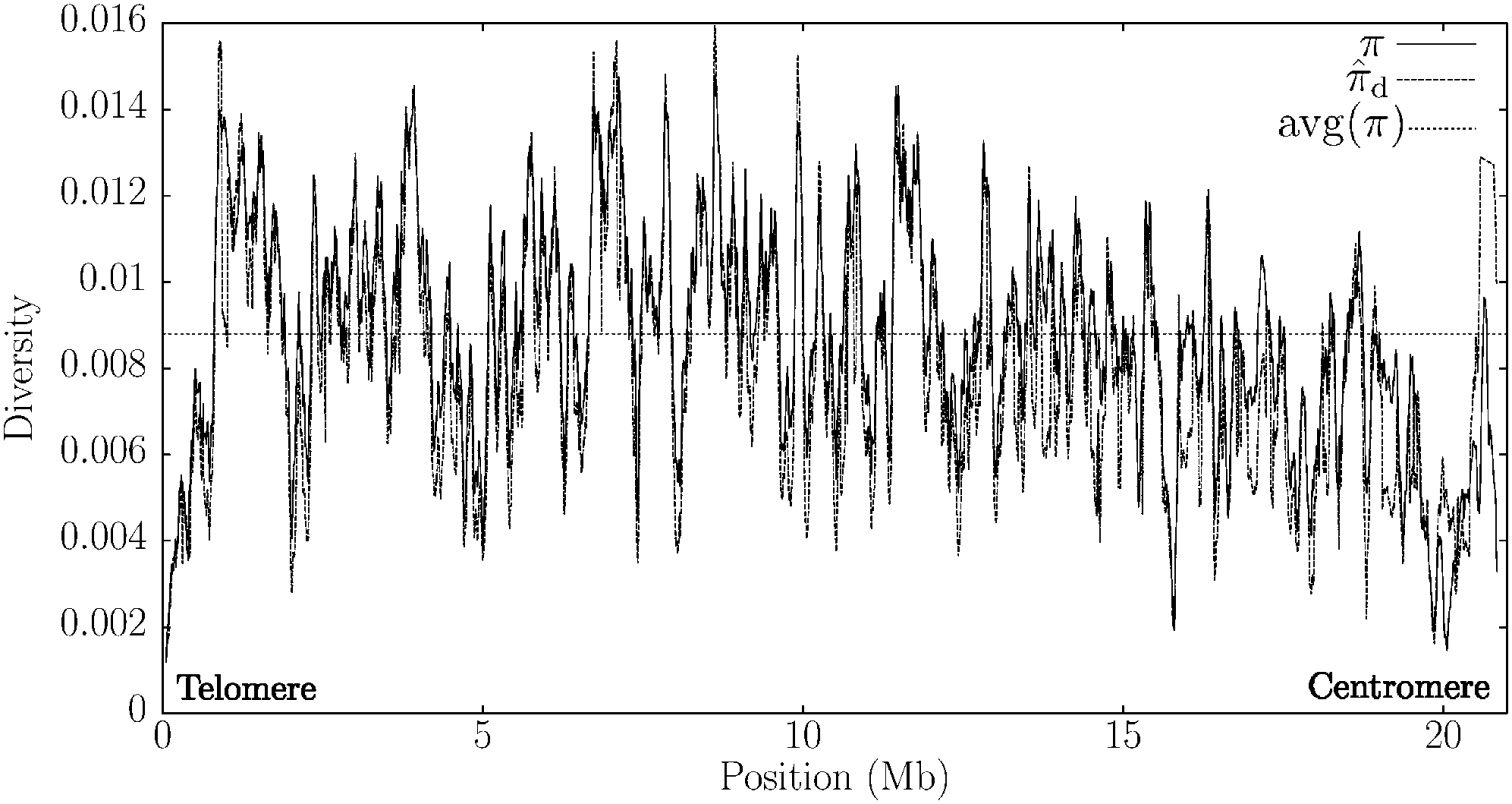
Comparison between the diversity measures *π* and ${\overset{⁁}{\pi }}_{\text{d}}$ along chromosome 2L of the two *Drosophila melanogaster* strains RAL-365 and RAL-391. Window length: 10^5^ bp, windows advanced by 10^4^ bp. In the ${\overset{⁁}{\pi }}_{\text{d}}$ analysis RAL-365 served as query.

To apply ${\overset{⁁}{\pi }}_{\text{d}}$ to real data sets, we compared two strains of *Drosophila melanogaster*, RAL-356 and RAL-391, whose genomes have been published as part of the Drosophila Population Genomics Project (www.dpgp.org). These sequences are distributed as alignments, which obviates an alignment-free approach. However, the existence of an alignment allows us to compare *π* with ${\overset{⁁}{\pi }}_{\text{d}}$, and Figure 6 shows a sliding window analysis for both quantities. With the exception of the centromeric region, ${\overset{⁁}{\pi }}_{\text{d}}$ appears to track *π* well. In particular, the well-known drop in diversity in the peritelomeric and pericentromeric regions is readily discernible. In the positions closest to the centromere, ${\overset{⁁}{\pi }}_{\text{d}}$ is consistently larger than *π*. One reason for this might be a lack of homologous sequence in strain RAL-391. This illustrates that low genetic diversity is diagnosed more reliably by our method than is high genetic diversity, which may result from missing data. To see this connection between missing data and overestimation of genetic diversity, imagine a region in the query sequence without homolog in the subject sequence. In that region, the shustrings would reflect short random matches, which mimics the short shustrings found in homologous regions with lots of mutations.

To limit the upward bias that can thus be introduced through missing data, we imposed a threshold on the number of distinct shustrings that can be reported for a given window as described under Approach and Data. If this threshold is exceeded, no ${\overset{⁁}{\pi }}_{\text{d}}$ value is returned for that window. Without this heuristic, ${\overset{⁁}{\pi }}_{\text{d}}$ would jump to 0.023 in the pericentromeric region (not shown), instead of the value of 0.013 reported by pid.

**Figure 7:**
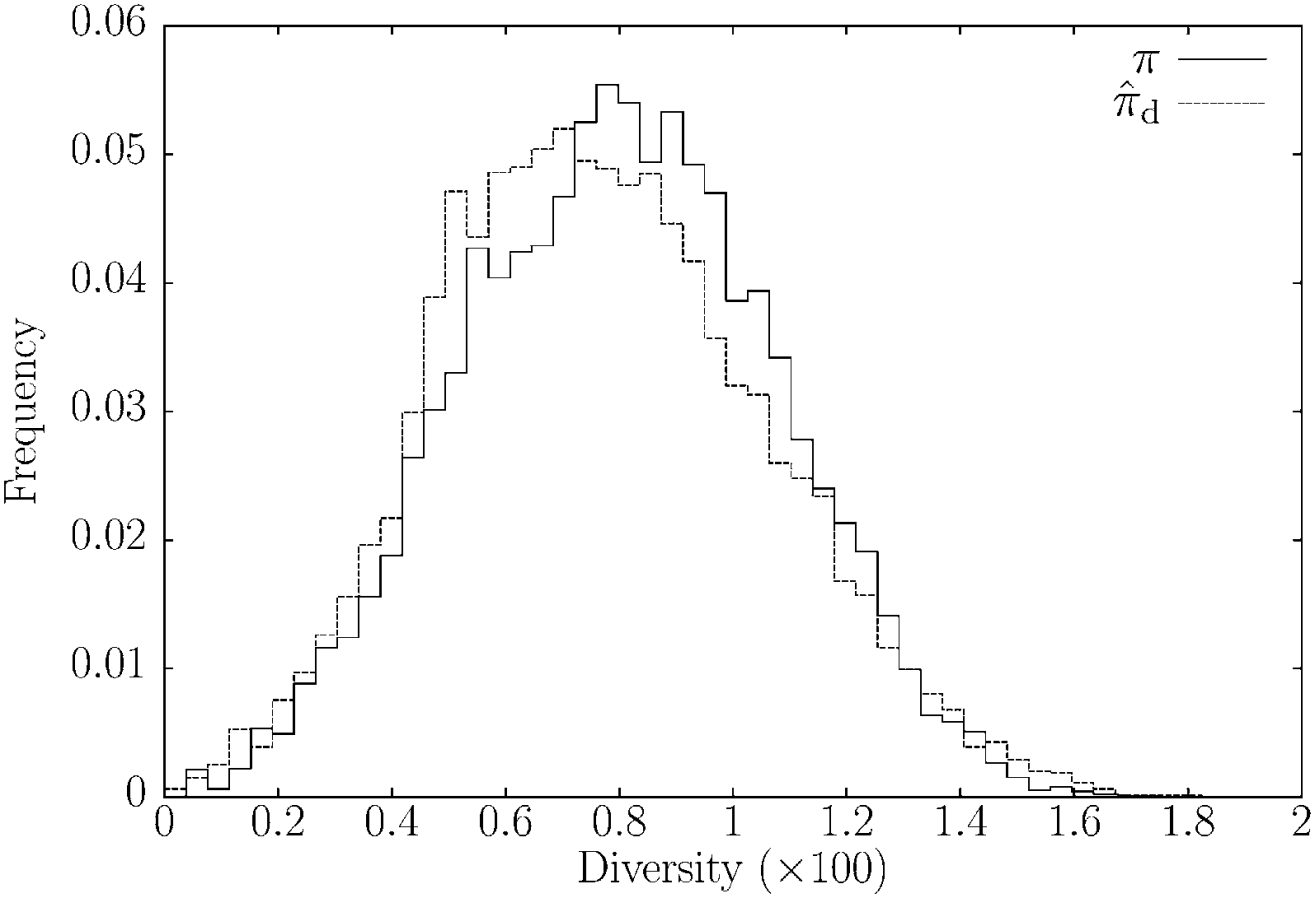
The frequency distributions of the diversity measures *π* and ${\overset{⁁}{\pi }}_{\text{d}}$ in 100 kb sliding windows along the full genomes of two strains of *Drosophila melanogaster*, RAL-365 and RAL-391.

In Figure 7 we compare the distribution of *π* with ${\overset{⁁}{\pi }}_{\text{d}}$ values across the entire genome. In spite of the problem with missing data just discussed, ${\overset{⁁}{\pi }}_{\text{d}}$ tends to be slightly smaller than *π*, which is reflected in the means of the two distributions, where $\text{mean}\left({\overset{⁁}{\pi }}_{\text{d}}\right)=0.0071$ is less than mean(*π*) = 0.0076.

**Figure 8:**
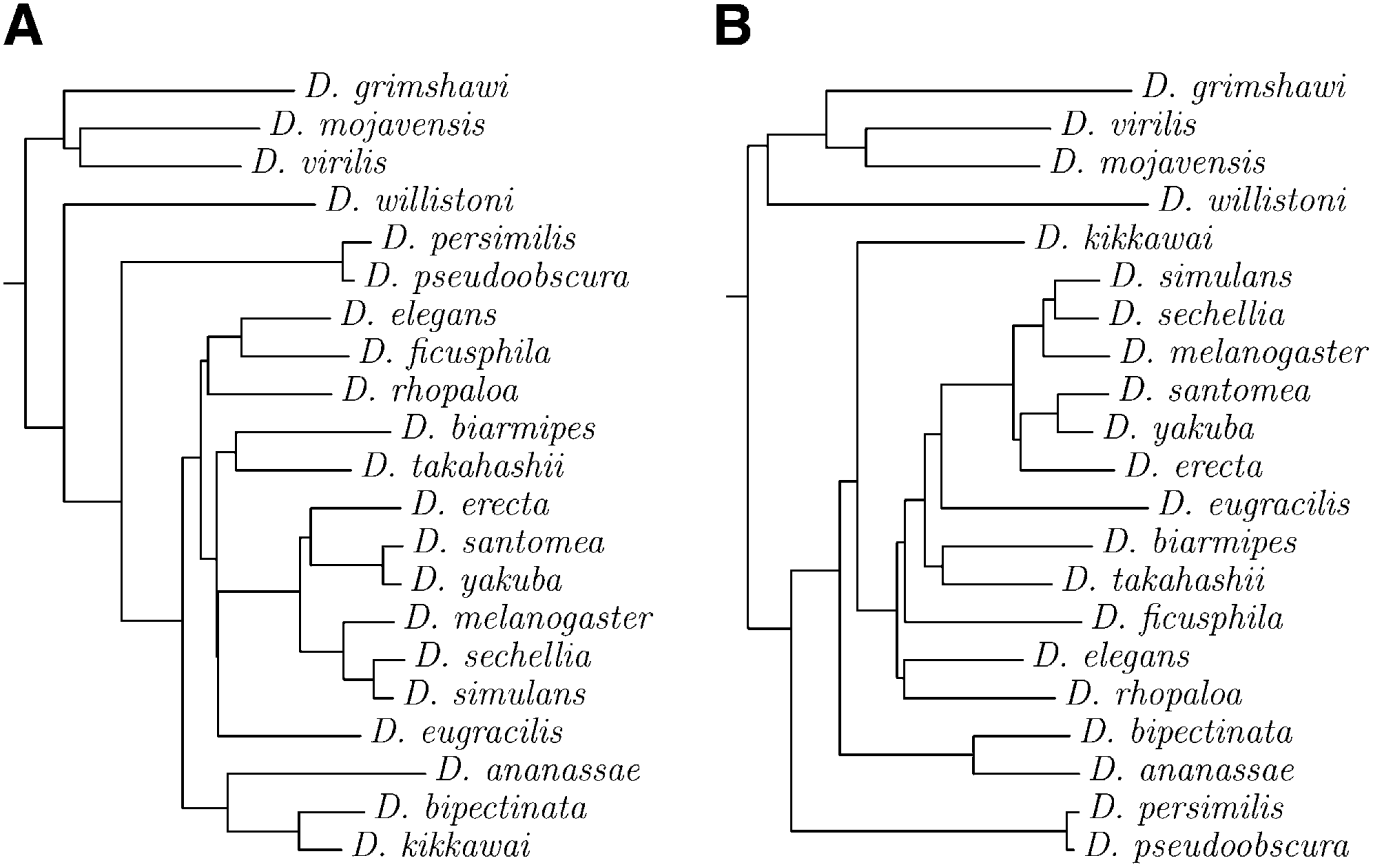
Neighbor-joining trees of the pairwise number of substitutions between 21 *Drosophila* species. (A) Substitution rates estimated without alignment from the full genome sequences. (B) substitution rates estimated from a multiple sequence alignment of the *Amyrel* gene.

To explore a pair of unaligned genomes, we turned our attention to the 21 complete genomes of *Drosophila* species currently available. Figure 8A shows a phylogeny of these species computed from their full genomes. For comparison, Figure 8B shows a corresponding alignment-based phylogeny computed just from the 2 kb sequences of the *Amyrel* gene (Da Lage *et al.* 2007). The two trees are reassuringly similar, especially for closely related clades.

We were looking for a pair of closely related genomes that had been assembled *de novo*. There were three candidate pairs: *D. sechellia*/*D. simulans*, *D. yakuba*/*D. santomea*, and *D. pseudoobscura*/*D. persimilis*. However, Haubold *et al.* (2011) had already investigated *D. sechellia*/*D. simulans*, and the genome of *D. santomea* appears to have been assembled on the scaffold of *D. yakuba*, yielding a pair of effectively aligned genomes. We therefore decided to compare the genomes of *D. pseudoobscura*/*D. persimilis*.

**Figure 9:**
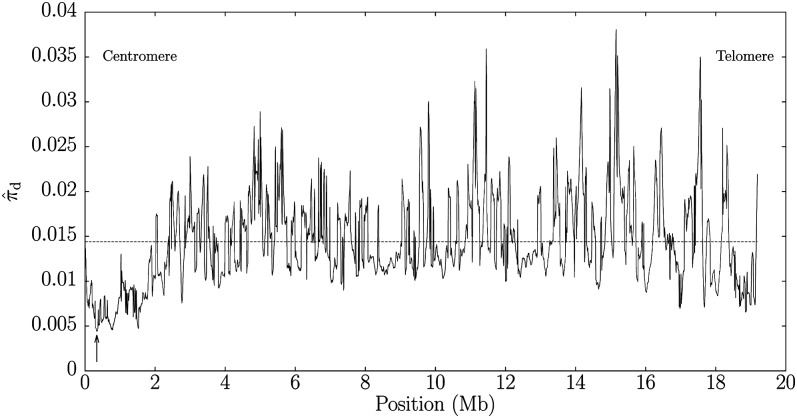
Sliding 100 kb windows along chromosome 3 of *Drosophila pseudoobscura* compared with *D. persimilis*. The arrow indicates the global minimum in genetic diversity between these two genomes, and the horizontal line the chromosome-wide average of ${\overset{⁁}{\pi }}_{\text{d}}$.

The genome of *D. persimilis* consists of 175.6 Mb distributed over 12,837 contigs, whereas that of *D. pseudoobscura* was largely made up of 15 contigs associated with chromosomes and a further 4025 unmapped contigs comprising 146.1 Mb in total. The sliding window comparison between *D. pseudoobscura* as query and *D. persimilis* as subject took 33 min on a a single AMD Opteron CPU running at 2.3 GHz. Chromosome 3 contains the genome-wide minimum in genetic diversity in its pericentromeric region. Figure 9 shows the location of this minimum among the fluctuating ${\overset{⁁}{\pi }}_{\text{d}}$ values along the length of chromosome 3. As previously observed by Noor *et al.* (2007), we find that the divergence is reduced not only in the pericentromeric region but also in the peritelomeric region.

## Discussion

Computation is the bridge between theory and experiment. The development of suitable computational methods has, therefore, been an integral part of population genetics for a long time. For example, Kingman's coalescent is, on the one hand, a mathematical concept (Kingman 1982), but when implemented as a computer program, it becomes an efficient tool for analyzing experimental data (Hudson 1983; Hudson 2002). More recent work on the ancestral recombination graph (Mcvean and Cardin 2005; Marjoram and Wall 2006) has lead to the very fast simulation program macs (Chen *et al.* 2009), to name but two examples of computational advances in population genetics.

Our development of an alignment-free diversity estimator, ${\overset{⁁}{\pi }}_{\text{d}}$, continues this tradition of applying mathematical or algorithmic discoveries to population genetics. Like most research on alignment-free algorithms, our work is motivated by efficiency considerations (Vinga and Almeida 2003). In situations of data super-abundance, a quick ${\overset{⁁}{\pi }}_{\text{d}}$ scan could be used to guide subsequent, more detailed alignment-based investigations.

There is a strong tradition of basing alignment-free sequence analysis on word counts as they can be computed easily (Reinert *et al.* 2009; Wan *et al.* 2010). In contrast, our method utilizes the computationally more involved distribution of shustring lengths along a genome. This distribution is similar to the match length distribution first investigated in DNA sequences by Arratia *et al.* (1986). These authors looked at the length distribution of random matches within a single sequence. In contrast, we compute match lengths between homologous pairs of sequences. The motivation for using this statistic is that it lends itself naturally to explicit evolutionary modeling, as it effectively deals with distances between SNPs.

The central feature of our model is the standard coalescent assumption that the time to the most recent common ancestor of two homologous sequence segments is exponentially distributed. Moreover, ${\overset{⁁}{\pi }}_{\text{d}}$ works not only for fully assembled genomes, but also for sets of contigs. The only two conditions imposed on the data are that (i) recombination is not much more frequent than mutation, and (ii) that edge effects can be neglected, in other words, that most shustrings end before the contig they appear in. This means that ${\overset{⁁}{\pi }}_{\text{d}}$ will be less precise if the data consists of many contigs rather than contiguous sequence, everything else being equal.

Algorithmically, ${\overset{⁁}{\pi }}_{\text{d}}$ is based on advances in string indexing, which allow fast lookup of shustring (shortest unique substring) lengths between genomes. To this preexisting technology we have added the derivation of the distribution of shustring lengths to arrive at a maximum-likelihood estimator of *π*, ${\overset{⁁}{\pi }}_{\text{d}}$. Recombination leads to fluctuating times to the most recent common ancestor along sequences, which is observable as clustered polymorphisms and an increase in the average shustring length. This effect of recombination on the average shustring length impaired the previous estimator of *π*, ${\overset{⁁}{\pi }}_{\text{m}}$, which was based on the assumption of constant coalescent times across the sequences compared (Haubold *et al.* 2011). By allowing the coalescent times to fluctuate and computing the new estimator ${\overset{⁁}{\pi }}_{\text{d}}$ from the whole distribution of shustring lengths (Figure 2), rather than just from their average, we have much improved the precision of our previous estimator (Figures 3 and 4), while keeping its implementation fast.

The advantage of the new approach is especially apparent in sliding window analyses, and Figure 5 demonstrates the accuracy of sliding ${\overset{⁁}{\pi }}_{\text{d}}$ when applied to simulated data. However, the analysis of the two strains of *Drosophila melanogaster* from North Carolina revealed a small downward bias of ${\overset{⁁}{\pi }}_{\text{d}}$. This might be due to recent gene duplications. These would lead to long shustrings and hence to an underestimation of *π*. Moreover, we assume independence between nucleotides, which is known not to apply in, for example, protein coding sequences or CpG islands. Higher order dependencies between nucleotides would also lead to longer shustrings than expected under our model and thereby to an underestimation of *π*. Finally, selection leads to longer haplotypes and concomitantly longer shustrings, which would also lower ${\overset{⁁}{\pi }}_{\text{d}}$.

The comparison of *D. pseudoobscura* with *D. persimilis* revealed a decrease in genetic diversity in the pericentromeric and the peritelomeric regions. Figure 9 clearly shows this valley in genetic diversity among the peritelomeric first 2 Mb of chromosome 3, which also contains the global diversity minimum. Such a reduction in genetic diversity at the ends of chromosome arms (Figure 6) is typical for intra-species comparisons among genomes of *D. melanogaster* (Begun *et al.* 2007). Noor *et al.* (2007) first observed that this is also present in the inter-species comparison between *D. pseudoobscura* and *D. persimilis*. They explained this as a remnant of the recent divergence of the species, leaving the well-known correlation between local diversity and recombination in *Drosophila* intact.

We plan to extend this work in two directions: First, we wish to develop an alignment-free test for recombination based on the fact that the mean shustring length is highly sensitive to recombination (Figure 3). Such a test might be useful for detecting recombination in bacterial genomes undergoing occasional horizontal gene transfer. Second, we plan to estimate diversity from samples of more than two sequences. Here, we would apply more specific properties of the coalescent to obtain an estimator of the population mutation rate *θ*, which could then be compared to Watterson's classical estimator (Watterson 1975).

We have shown that pid can be used to quickly compare genomes consisting of unmapped contigs. Unmapped contigs are difficult to align under the best of circumstances, but they increasingly form the end-result of genome sequencing efforts. Analysis of such data with pid could be an early step followed by more detailed investigations using alignment-based methods. Therefore, our alignment-free method is best viewed as complementary to alignment-based approaches whenever a rough and ready prescreening of population genomics data is desired. However, in spite of the simplifying assumptions we have made, our method is accurate enough to reveal the diversity landscape along metazoan chromosomes.
